# *#TheDress*: The Role of Illumination Information and Individual Differences in the Psychophysics of Perceiving White–Blue Ambiguities

**DOI:** 10.1177/2041669516645592

**Published:** 2016-04-28

**Authors:** Vera M. Hesslinger, Claus-Christian Carbon

**Affiliations:** Department of General Psychology and Methodology, University of Bamberg, Bamberg, Germany; Abteilung Allgemeine Experimentelle Psychologie, Johannes Gutenberg University, Mainz, Germany; Department of General Psychology and Methodology, University of Bamberg, Bamberg, Germany; Bamberg Graduate School of Affective and Cognitive Sciences (BaGrACS), Germany

**Keywords:** Ambiguity, color constancy, color perception, *#TheDress*, indeterminacy

## Abstract

In early 2015, a public debate about a perceptual phenomenon that impressively demonstrated the subjective nature of human perception was running round the globe: the debate about *#TheDress,* a poorly lit photograph of a lace dress that was perceived as white–gold by some, but as blue–black by others. In the present research (*N* = 48), we found that the perceptual difference between *white–gold perceivers* (*n*_1_ = 24, 12 women, *M*_age_ = 25.4 years) and *blue–black perceivers* (*n*_2_ = 24, 12 women, *M*_age_ = 24.3 years) decreased significantly when the illumination information provided by the original digital photo was reduced by means of image scrambling (Experiment 1). This indicates that the illumination information is one potentially important factor contributing to the color ambiguity of #TheDress—possibly by amplification of a slight principal difference in psychophysics of color perception which the two observer groups showed for abstract uniformly colored fields displaying a white–blue ambiguity (Experiment 2).

In early 2015, a public debate about a perceptual phenomenon that impressively demonstrated the subjective nature of human perception was running round the globe: the debate about *#TheDress*. It had been initialized by the photo of a lace dress posted on Tumblr (http://swiked.tumblr.com/post/112073818575/guys-please-help-me-is-this-dress-white-and; last accessed September 26, 2015, 6:54 p.m. CMT+1, unfortunately, the original link is not available anymore; alternatively see: https://en.wikipedia.org/wiki/The_dress_%28viral_phenomenon%29) that evoked ambiguous color perceptions in its observers. The debate mainly went: *It’s white with golden lace!—No, it’s blue and black!—No, no, now it’s this, then it’s that, I can switch!* … The observation that a stimulus can induce intra- or interindividually varying, sometimes even switching perceptions is not new, indeed. Perceptual multistability elicited by ambiguous figures such as the Necker cube is a classic example in this regard, and the evocation of perceptual challenge by means of ambiguity is even a core aesthetic principle, for example, in modern art (Muth, Hesslinger, & Carbon, 2015). Concerning color perception, intra- or inter-individual variation is known as well: Even though the human visual system is equipped with mechanisms to preserve color constancy (e.g., Gegenfurtner, 2003), our perceptions of an object’s color can vary, for instance, as these mechanisms work more or less well depending on illumination condition and background (Pearce, Crichton, Mackiewicz, Finlayson, & Hurlbert, 2014). Interobserver discrepancies in perceived color can further be related to factors such as differing current states of chromatic adaptation, different color memories of observers encountering an object, and differences in the composition of retinal photo pigments (e.g., Jameson, Highnote, & Wasserman, 2001; Mollon, 1992).

In order to explain the differently perceived colors of *#TheDress*, several assumptions have been brought forward by vision scientists, for example, that the different perceptions were related to differential color sensitivity or differences in anchoring and the resulting lightness perception (cf. Gilchrist et al., 1999). In the present research, we focussed on the hypothesis that interindividual differences in the use of illumination information (e.g., Palmer, 1999) contribute to the variance in the perception of #TheDress. This hypothesis or, as Brainard and Hurlbert (2015) called it, the “colour constancy explanation” refers to the mechanism which enables color constancy under varying illumination conditions by inferring the type or quality of illumination of a scene and correcting the percept accordingly. With regards to #TheDress, it was hypothesized that some persons infer and correct for a bluish illumination, thus perceiving a white–golden dress, while others infer and correct for a yellowish illumination, thus perceiving a blue–black dress.

## General Method

The sample, setting, and apparatus were the same for both experiments. We describe them here in the first part. Detailed information on the procedure and results of each experiment will follow straight afterwards in the respective sections.

### Participants

A total of 48 volunteers (24 women; *M*_age_ = 24.8 years) with normal or corrected-to-normal vision (checked via Snellen test) and normal color vision (checked via a short version of the Ishihara test) participated in the study which comprised Experiments 1 and 2. Each participant completed both experiments within one session. In order to prevent the psychophysical functions assessed in Experiment 2 from being biased by previous exposure to the photo of #TheDress which was shown in Experiment 1, the order the experiments were actually executed in was inverse to the numbering we use here: first Experiment 2, then Experiment 1. On basis of the ratings participants gave for the original digital photo of #TheDress in Experiment 1, they were post-hoc classified as *white–gold perceivers* and *blue–black perceivers*, respectively. The rating scale ranged from 0 (*not blue at all*) to 100 (*very blue*), and participants’ classification was executed on basis of a mean score of 50*—*actually the result of classification would have been exactly the same if we had used a median split instead as the median was 52.5, so very close to the mean score of 50. By chance, it turned out that half of the sample was to be classified as white–gold perceivers (*n_1_* = 24, 12 women; *M*_age_ = 25.4 years), and the other half was to be classified as blue–black perceivers (*n_2_* = 24, 12 women; *M*_age_ = 24.3 years). The two groups clearly differed from each other (*M*_diff_ = 42.9, *t*_46_ = 9.7, *p* < .0001, Cohen’s *d* = 2.80—a very large difference regarding Cohen’s definition of a *d* = 0.80 as indicating an already large effect). Participants were naive to the purpose of the experiments and were tested individually. All procedures were in accordance with the national ethical standards on human experimentation and with the Declaration of Helsinki of 1975, as revised in 2008. Informed written consent was obtained from all participants.

### Setting and Apparatus

Both experiments were conducted in the same laboratory with constant lighting conditions (no daylight entering from outside; illumination of the lab via Osram cool daylight technology). The experiments were run on a Dell Desktop PC with a true color Eizo Color Graphic CG245W TFT monitor (wide screen; 24.1″ diagonal, resolution: 1,920 × 1,200 pixels, color depth: 32 bits per pixel, refresh rate: 60 Hz). The monitor was linearized via the integrated calibration routine (IPS Panel) at the beginning of each session. The white point of the monitor was *x*, *y* = 0.3324, 0.3474 (D55; 5497 K); the empirical primaries of our monitor were Red (R) = (0.6767, 0.3068, 27.65), Green (G) = (0.1998, 0.6860, 64.32), and Blue (B) = (0.1507, 0.0537, 5.24) in terms of Commission Internationale de l’Éclairage (CIE) color space xyY values. During the experiments, the participants were sitting at a constant distance of 50 cm in front of the screen, which was ensured by using a chin–forehead rest. For stimulus delivery and data collection we used the up-to-date Experiment Builder version 1.10.1241 (SR Research Ltd., Mississauga, Ontario).

## Experiment 1

In Experiment 1, we tested whether illumination information provided by the photo of #TheDress had an impact on the perception of the colors of #TheDress. Our rationale was: If the color constancy explanation (see earlier text) is right, that is if interindividual differences in the use of illumination information significantly contribute to the differences in the perception of the dress, these differences will decrease when the illumination information of the image is reduced. A reduction of illumination information was implemented by means of image scrambling.

### Method

#### Stimuli

**Figure 1.**
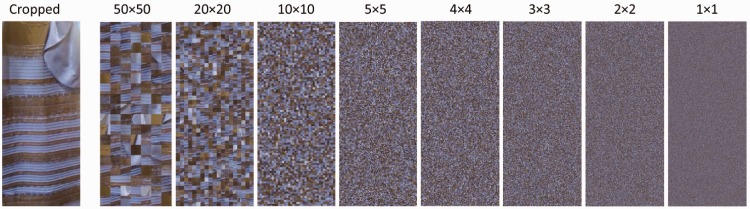
Selection of stimuli used for testing the impact of illumination information; cropped image rectangle selected from the original digital photo (leftmost) and several scrambled variants with decreasing side length of scramble squares (i.e., increasing segmentation) from left to right. Numbers indicate the side lengths in horizontal × vertical pixels.

We used the original digital photo of #TheDress and multiple variants of the same that varied with regards to the degree of information about illumination. Photographs usually provide some kind of information about the illumination of the original scene such as background or surround, detectable shadows, reflections, familiar objects the color of which is known, and so forth. For the present experiment, we systematically reduced illumination information by cropping and scrambling the digital photograph of #TheDress that had been posted on tumblr. More precisely, we first selected a rectangular part of the original digital photo so that there was nothing of the original background left in the resulting cropped image rectangle to prevent integrative scrambling of the background (see Figure 1, leftmost depiction). Then we scrambled the cropped image rectangle (384 × 904 pixel, visual angle 11.8° × 27.4°) several times using different sizes of scramble squares. The scrambling process was executed via a self-programmed Matlab routine that segmented the image into scramble squares with side length *a* which were randomly permuted. For the present experiment, we defined the following 17 side lengths: *a* = 200, 150, 100, 90, 80, 70, 60, 50, 40, 30, 20, 10, 5, 4, 3, 2 pixels, and 1 pixel. The smaller the used scramble squares were (i.e., the more “segmented” the image was), the less valid illumination information was preserved in the stimulus. So, for the scrambled version with the smallest scramble squares, illumination information was most strongly reduced as compared with the original photo (though not fully abolished, of course). The scrambling process was performed twice for each side length. We thus obtained 2 × 17 = 34 scrambled versions. In sum, we had 1 (original digital photo of #TheDress) + 1 (cropped image rectangle) + 34 (scrambled versions of the cropped image rectangle) = 36 stimuli to be used in Experiment 1. Several exemplary stimuli are presented in Figure 1 (*Note*. Scrambling is only one possible procedure for systematically reducing the degree and validity of illumination information besides, for example, phase manipulation by adding noise, see Wichmann, Braun, & Gegenfurtner, 2006. We decided to use scrambling as it preserves the local color information of the image).

#### Procedure

**Figure 2.**
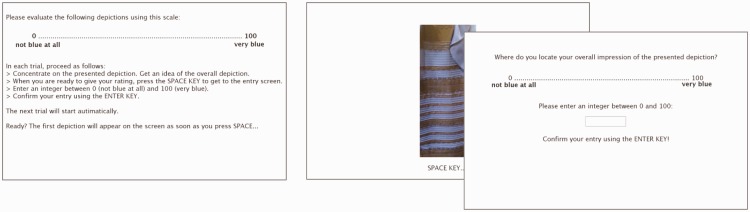
General instructions (left) and trial scheme (right) for Experiment 1; wording translated (original wording was German).

Participants rated the original digital photograph of #TheDress as well as a cropped rectangular version and scrambled variants of the same with regards to their degree of being blue. The whole experiment comprised 36 trials. In each trial, one of the 36 stimuli was presented singly in the center of the screen until participants pressed the space key to indicate that they were ready to give their rating. On the following screen, participants were asked to indicate their impression of how blue the just-presented stimulus was. In order to do so, they entered an integer between 0 = *not blue at all* and 100 = *very blue* and confirmed their decision by pressing the enter key. The next trial started automatically (inter trial interval [ITI] = 1,200 ms). The order of stimulus presentation was randomized. Instructions and a trial scheme for Experiment 1 are shown in Figure 2.

### Results and Discussion

**Figure 3.**
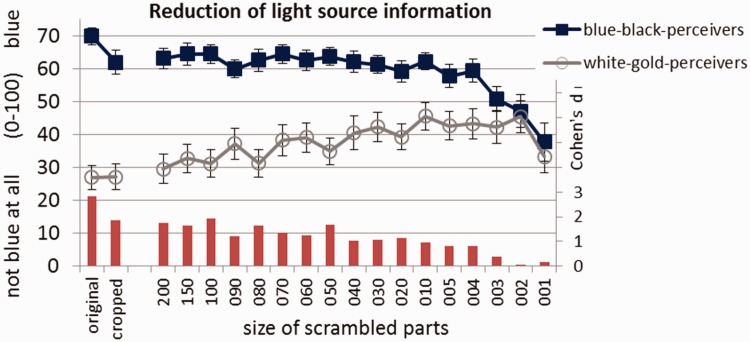
Perceived degree of “not blue at all” (i.e., toward white)/“blue” for the different scrambled variants split by perceiver group. Numbers on the abscissa indicate the side-length of the scramble squares; the smaller the side-length of the squares, the stronger the segmentation of the image and the reduction of illumination information. Error bars indicate ± 1 SEM. All differences between the perceiver groups are significant (with Bonferroni-corrected α) except for side-lengths 001 to 005. Red bars indicate effect sizes (Cohen’s *d*).

While white–gold perceivers and blue–black perceivers differed markedly in their interpretations of the color of less segmented versions, their ratings became closer and closer to each other with increasing image segmentation (Figure 3). In fact, the difference between both groups’ interpretations of the version that was segmented to a very high degree (where scramble squares of very small side length were used) and thus provided the least illumination information was statistically insignificant (see also caption of Figure 3). The pattern of results we found indicates that the (specific) background and illumination of the original digital photo of #TheDress contribute to the variation in the perception of the colors of the depicted garment. This is in line with some of the findings of Lafer-Sousa, Herman and Conway (2015), who showed that perceivers vary more in their judgments of the colors of #TheDress when the photo is presented with the original background than when it is presented with alternative backgrounds that provide “unambiguous illumination cues” (p. R2).

The next question, however, is: Which factor(s) might underlie this individual difference in color constancy found for #TheDress? Brainard and Hurlbert (2015) suggested testing for other individual differences using stimuli that are much simpler than #TheDress (p. R553). That was exactly what we did in the second experiment.

## Experiment 2

In Experiment 2, we investigated whether white–gold and blue–black perceivers also differ in their perceptions of simpler stimuli. To that end, we assessed the different observers’ psychophysical functions of color perception for a white–blue and a gold–black color continuum, respectively, and compared the perceptions of the two observer groups (white–gold and blue–black perceivers).

### Method

#### Stimuli

We defined two color continua, one ranging from white (RGB 255, 255, 255; i.e., CIE^1^ xyY: 0.3340, 0.3456, 98.68) to blue (RGB 0, 0, 255; i.e., CIE xyY: 0.1507, 0.0537, 5.24), and one ranging from gold (RGB 255, 215, 0; i.e., CIE xyY: 0.4761, 0.4653, 76.57) to black (RGB 0, 0, 0; i.e., CIE xyY: 0.2842, 0.3158, 0.30), respectively—see General Method for monitor primaries. We divided each continuum into 51 color levels (shades) to be presented on the screen in the form of uniformly colored full-screen rectangles (1,920 × 1,200 pixel, visual angle 54.6° × 35.9°).

#### Procedure

**Figure 4.**
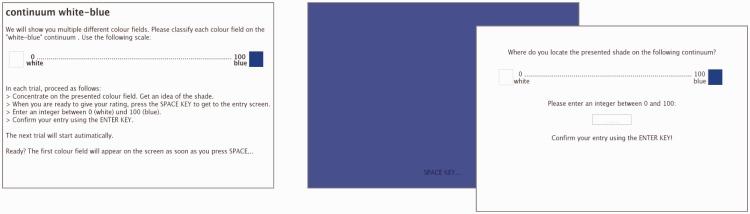
General instructions (left) and trial scheme (right) for Experiment 2/part 1 (white–blue continuum); wording translated (original wording was German).

The experiment comprised two parts. In the first part, participants rated all 51 color levels of the white–blue continuum; in the second part, they rated all 51 color levels of the gold–black continuum. For each continuum, the respective stimuli were presented on the screen one by one in randomized order until participants pressed the space key to indicate that they were ready to give their rating. On the following screen, participants were asked to indicate the color of the presented stimulus by entering an integer between 0 and 100 (part 1: 0 = *white*, 100 = *blue*; part 2: 0 = *gold*, 100 = *black*) and to confirm their decision by pressing the enter key. The next trial started automatically (inter trial interval ITI = 1,200 ms). In sum, the experiment comprised 2 (parts) × 51 (stimuli) = 102 trials. Instructions and a trial scheme for the white–blue continuum are shown in Figure 4.

### Results and Discussion

**Figure 5.**
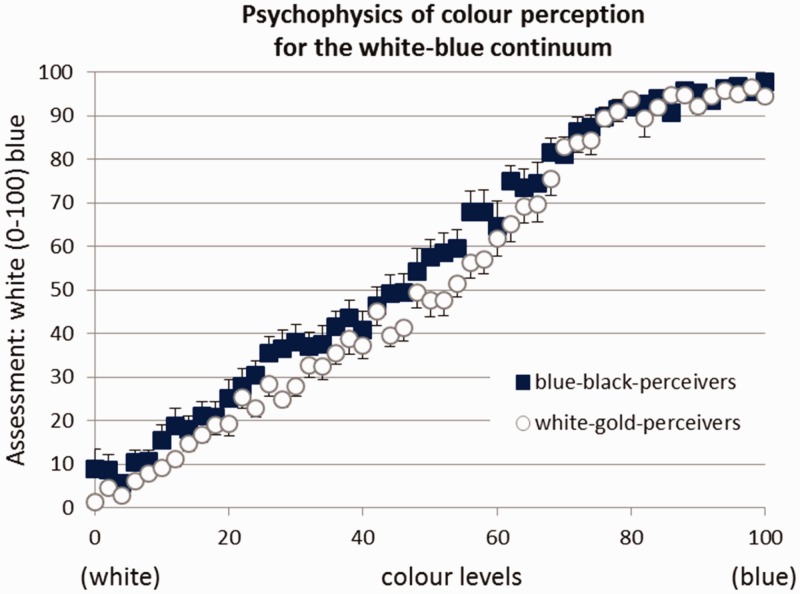
Psychophysics of color perception for the white–blue continuum split by perceiver group. Error bars indicate ± 1 SEM.

On the white–blue but not the gold–black continuum, the average psychophysical function obtained for white–gold perceivers differed slightly but systematically and significantly from the function obtained for blue–black perceivers (*p* = .0434, η*_p_*^2^ = .092), especially in the case of color levels (shades) that were colorimetrically more white than blue. Here, blue–black perceivers reported higher amounts of blue than white–gold perceivers (see Figure 5; although interaction color level × perceiver group was *not* reaching the significance level of .05, concretely: *p* = .0663, the systematic difference between both groups along a wide range of color levels was quite obvious although being quite small in size). This might reflect a slightly higher sensitivity for blue components or, at least, a slight bias toward interpreting white–blue ambiguities as blue rather than white.

## General Discussion

The divergent perceptions of #TheDress did also show up under highly standardized lab conditions with a color-calibrated monitor (see also, e.g., Gegenfurtner, Bloj, & Toscani, 2015). This demonstrates that the observed variance in perceiving #TheDress is not merely a result of different technical presentation devices used by different observers in the field.

In the present study, we tested the so-called “colour constancy explanation” (Brainard & Hurlbert, 2015) that explains the variation in the perceptions of #TheDress on basis of a specific mechanism involved in obtaining color constancy under different illumination conditions. In Experiment 1, we found that the illumination information provided by the original digital photo of #TheDress and the way it is used by the observer seems to be one potentially important factor for the divergence it evokes, indeed. Our results may support the assumption that white–gold and blue–black perceivers process or interpret this information differently. This difference between the two observer groups might trace back to and amplify a more basic interindividual difference in the psychophysics of perceiving stimuli that “display” a color ambiguity on the white–blue continuum. In Experiment 2, we found that observers who perceive #TheDress as blue and black are a little bit more ready to interpret or perceive such ambiguity as blue rather than white. Interestingly, for the gold–black continuum, we did not find a significant difference between the psychophysical curves of the two observer groups. This indicates that the interpretation and perception of colors on the white–blue continuum are of specific importance here.

It should be noted that our results are not only compatible with the color constancy explanation. For instance, the scrambling method utilized in Experiment 1 does not only reduce illumination information, but also disrupts form, so Gestalt detection is undermined and, ultimately, the meaning of the image is not (easily) accessible any longer. The Gestalt itself, in this case the dress, its design and the texture of the material it is made of, however, are potential cues for inferences about color. Lafer-Sousa et al. (2015), for example, observed that relatively more observers interpreted #TheDress as being white and golden, when the photo was presented at larger sizes so that high-spatial frequency information, which provides a cue to the material, was more evident.

In our study, the participants did not explicitly report changes of their percepts of #TheDress. Yet, in related studies in which we investigated further aspects of the phenomenon of #TheDress in different contexts (unpublished), we registered so-called “switchers” as well, and according to the data of Lafer-Sousa et al. (2015), for instance, numerous naive observers (45%) experience a switch between the first and following exposures. How to relate such cases of switching to our results on (static) group differences in psychophysics of interpreting and perceiving white–blue ambiguities is a question that is not easily answered, but requires further empirical investigation.

The difference between the psychophysical curves of white–gold and blue–black perceivers that we found in Experiment 2 might be a first indication of more basic differences underlying the phenomenon of different percepts observed for #TheDress. An important next step would be locating the (physiological) basis of the found psychophysical results within the visual apparatus. Furthermore, the processes relating them to the differential use of illumination information remain to be identified.

## Note

1 Via the experimental program we set the colours as RGB values, but measured the consequential CIE xyY values via the monitor-integrated IPS Panel.
